# Ability of Non-Hosts and Cucurbitaceous Weeds to Transmit Cucumber Green Mottle Mosaic Virus

**DOI:** 10.3390/v15030683

**Published:** 2023-03-04

**Authors:** David A. Lovelock, Sharl J. L. Mintoff, Nadine Kurz, Merran Neilsen, Shreya Patel, Fiona E. Constable, Lucy T. T. Tran-Nguyen

**Affiliations:** 1 Department of Jobs Precincts and Regions, Agriculture Victoria Research, Agribio, Melbourne, VIC 3083, Australia; fiona.constable@agriculture.vic.gov.au; 2 Department of Industry, Tourism and Trade, Biosecurity and Animal Welfare, Darwin, NT 0801, Australia; sharl.mintoff@nt.gov.au (S.J.L.M.); nadinekurz@googlemail.com (N.K.); merran.neilsen@gmail.com (M.N.); shreya.patel@nt.gov.au (S.P.); 3 Plant Health Australia, Level 1, 1 Phipps Close, Canberra, ACT 2600, Australia; ltran-nguyen@phau.com.au

**Keywords:** Cucumber green mottle mosaic virus, weeds, non-hosts

## Abstract

Cucumber green mottle mosaic virus (CGMMV) is a *Tobamovirus* of economic importance affecting cucurbit crops and Asian cucurbit vegetables. Non-host crops of CGMMV, including capsicum (*Capsicum annum*), sweetcorn (*Zea mays*), and okra (*Abelmoschus esculentus*), were tested for their susceptibility to the virus, with field and glasshouse trials undertaken. After 12 weeks post-sowing, the crops were tested for the presence of CGMMV, and in all cases, no CGMMV was detected. Commonly found within the growing regions of cucurbits and melons worldwide are weeds, such as black nightshade (*Solanum nigrum*), wild gooseberry (*Physalis minima*), pigweed (*Portulaca oleracea*), and *Amaranth* species. Several weeds/grasses were tested for their ability to become infected with CGMMV by inoculating weeds directly with CGMMV and routinely testing over a period of eight weeks. *Amaranthus viridis* was found to be susceptible, with 50% of the weeds becoming infected with CGMMV. To further analyse this, six Amaranth samples were used as inoculum on four watermelon seedlings per sample and tested after eight weeks. CGMMV was detected in three of six watermelon bulk samples, indicating that *A. viridis* is a potential host/reservoir for CGMMV. Further research into the relationship between CGMMV and weed hosts is required. This research also highlights the importance of proper weed management to effectively manage CGMMV.

## 1. Introduction

Cucumber green mottle mosaic virus (CGMMV) (genus *Tobamovirus*) infects melon and cucurbit hosts along with several Asian cucurbit vegetables. The virus was first identified in 1935 [1] and has been detected in several countries, including Israel, Canada, the USA, and more recently, from multiple detections in Australia from 2014 [2,3,4,5]. Losses associated with CGMMV infection can be detrimental, through the loss of already planted crops and the potential inability to re-plant for many months. However, its ability to infect outside of *Cucurbitaceae* hosts is still not fully understood. Viruses of the *Tobamovirus* genus, including tobacco mosaic virus, pepper mild mottle virus, and tomato mosaic virus, are known to infect several weed species and non-*Curcurbitaceae* crop species. [6,7]. Similarly, yellow tailflower mild mottle virus has been shown to infect *Solanum nigrum* (black nightshade) and *P. peruviana* (cape gooseberry) [8,9].

A 2016/2017 survey of cucurbit growing regions in Australia documented the weeds that are commonly found, which include fat hen (*Chenopodium album*), pigweed (*Portulaca oleracea*), Amaranth species, and blackberry nightshade (*Solanum nigrum*) [10]. The presence of these weed species and their susceptibility to CGMMV has previously been studied in 2004 [11] and 2017 [3], with indications that weeds such as fat hen and pigweed are hosts of the virus. In the 2017 [3] study, the following weeds were all found to be susceptible to CGMMV: *Moluccella laevis*, *Amaranthus graecizans*, *A. muricatus*, *Chrozophora tinctoria*, *Withania somnifera,* and *Physalis somnifera.* However, the ability of these weeds, and others that are known hosts, to successfully transmit the virus to other *Cucurbitaceae* hosts has not been well documented. Previous research in Brazil in watermelon growing regions has identified several weeds, including *A. spinosus*, *P. angulate,* and *Heliotrpoium indicum*, as potential reservoirs for viruses of a range of genera, including potyviruses, cucumoviruses, and orthotospoviruses [12].

Following the initial detection of CGMMV in the Northern Territory (NT) in 2014, no host plants were permitted to be grown on the properties, and potential weed hosts were eradicated. Quarantine measures for CGMMV in the NT were lifted in February 2016. The inability of cucurbit growing regions to continue to produce cucurbit crops is of concern for the ongoing viability of farms. There is evidence of using non-host crops, such as rice and radish, as a means of reducing the impact of CGMMV [13].

This study aims to provide information on potential alternative non-host crops by investigating the interaction between several crops and CGMMV, in both field and screenhouse settings. The use of these non-hosts may provide an alternative income, whilst potentially reducing the inoculum load in the soil, allowing for future cucurbit production. A second aim is to further investigate the role of common weeds found in cucurbit growing regions, and their potential to harbor the virus and to serve as sources of infection for cucurbit crops. The information gained from studying these weeds may help in the management of CGMMV, and further the need for weed maintenance during the growing seasons.

## 2. Materials and Methods

### 2.1. Growth and Maintenance of Non-Hosts and Weeds

The non-host plants chosen for this study: capsicum (*Capsicum annum*), okra (*Abelmoschus esculentus)*, sweetcorn (*Zea mays*), sorghum (*Sorghum bicolor*), snake bean (*Vigna unguiculata* ssp. *Sesquipedalis*), and peanut (*Arachis hypogaea*), were supplied by a commercial nursery (PlantSmith Nursery, Darwin, NT, Australia) for all field and screenhouse trials. Weeds and weed seeds (Amaranth (*Amaranthus viridis*), pigweed (*Portulaca oleracea*), black nightshade (*Solanum nigrum*), wild gooseberry (*Physalis angulata*), fat hen (*Chenopodium album*), and Sabi grass (*Urochloa mosambicensis*)) were collected opportunistically, allowing the production of more seeds through fruiting bodies. All plants and weeds were tested for CGMMV using the methods stated in this method, prior to the trials commencing, ensuring that positive detections occurred only through manual inoculation.

### 2.2. Non-Host Field Site Preparation

A secure and fully enclosed fenced paddock field trial was set up at the Berrimah Farm Science Precinct at the Department of Industry, Tourism and Trade, Northern Territory, with field beds, irrigation tape, and mulch film installed. Prior to the irrigation tape and mulch film being laid, CGMMV-infected material was tilled into the soil to increase the chance of inoculum contacting the roots of the crops. Roughly 80 sweetcorn, capsicum, and sorghum seedlings were planted into the field beds, while 80 peanuts were sown.

### 2.3. Positive CGMMV Material Confirmation for Inoculations

Material collected during the 2014/2015 surveillance period for CGMMV was tested for CGMMV using Sanger sequencing, with the closest match resulting in > 99% sequence similarity to a partial CGMMV sequence from the Northern Territory (KM363234) [2]. This material was used to inoculate either tobacco or watermelon seedlings to provide bulk material for plant bioassay experiments. Prior to inoculation of all plant material for each trial, the material was confirmed to be positive for CGMMV using the assays described in the Methods section, with all positive test plants and a selection of the positive plants Sanger sequenced, with all results > 99% sequence similarity to the original inoculum.

### 2.4. Screenhouse Trials

**Figure 1:**
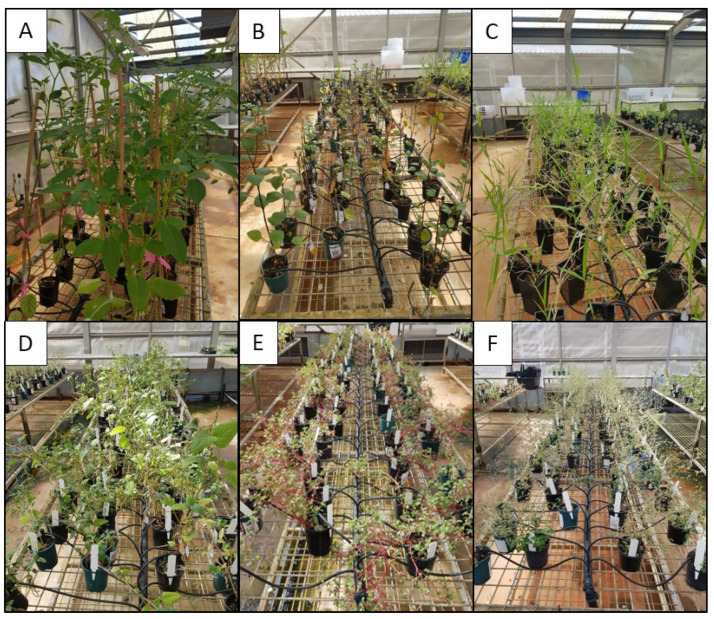
Weeds grown in screenhouse and tested for susceptibility to cucumber green mottle mosaic virus: (**A**) wild gooseberry, (**B**) black nightshade, (**C**) Sabi grass, (**D**) Amaranth, (**E**) pigweed, and (**F**) fat hen.

The secure screenhouse at Berrimah Farm Science Precinct was outfitted with automatic watering, with 80 drippers installed per bench, and five benches in total. The following crops were tested for susceptibility to CGMMV: *S. bicolor*, *A. hypogaea*, *A. esculentus*, *C. annum*, *Z. mays,* and *V. unguiculata ssp. Sesquipedalis*. The following weeds were also selected for susceptibility to CGMMV: *A. viridis*, *P. oleracea*, *C. album*, *U. mosambicensis*, *S. nigrum,* and *P. angulata* (Figure 1). 

In 100 mm pots, potting mix produced at Berrimah Farm Science Precinct, Coir chip/coir fines/fine pine bark (50%/35%/15%), was added to 80 pots per crop. One crop per pot was then planted into the potting mix with a total of 80 pots per crop, and the crops were inoculated with CGMMV two days later. Briefly, positive material was dried and roughly chopped. A phosphate-buffered saline (PBS) solution and a small amount of silica carbide (Sigma-Aldrich, St Louis, MO, USA) was added to the material. The inoculum was then roughly rubbed onto the leaves, allowing for a small amount of damage to increase the chances of the plants becoming infected. Inoculum (~750 µL) was added directly to the soil. Inoculated leaves were tagged with white marking tags on light string to ensure these were not sampled later.

One week later, 80 cucurbit plants were placed into pots of the same size with potting mix and manually inoculated with CGMMV as a positive control, and placed on a separate bench with automatic watering. Another 80 cucurbit plants used as negative controls were potted and placed on a bench and hand watered. 

Plants were left to grow for 8 weeks. Prior to sampling, material was disposed of and the screenhouse was decontaminated. A sample size of 80 was chosen, as this number of replicates could be undertaken in both a field and screenhouse setting in the space provided, with enough space between plants to prevent cross contamination. The number of samples selected would also statistically allow for greater confidence in the results, with presence/absence being tested for.

### 2.5. Isolation of Viral RNA, PCR Primers, and Conditions

Bulk samples (1 leaf per plant, 10 plants in total) were collected from each sample and roughly chopped, and a subsample of the material was placed into 12 cm × 15 cm Bioreba^®^ (ThermoFisher Pty Ltd., Waltham, MA, USA) extraction bags. Total RNA was then extracted using the Isolate II Plant RNA Kit (Bioline Pty Ltd., Taunton, MA, USA) according to the manufacturer’s instructions, with the following modification made: 700 µL of RLY Buffer and 7 µL of β-mercaptoethanol were added to the extraction bag, and the material was then ground before applying the liquid to the first column.

Conventional RT-PCRs were performed using a Veriti Thermal Cycler (ThermoFisher Scientific, Waltham, MA, USA), utilising Superscript III One Step with Platinum *Taq* (ThermoFisher Scientific, Waltham, MA, USA), following the manufacturers guidelines. The conditions used in this study can be found in a recent publication from the author [14], and the primers used were: Coat Protein (CP) (496 bp) [15] Forward primer, 5′-GATGGCTTACAATCCGATCAC-3′ and Reverse primer, 5′-CCCTCGAAACTAAGCTTTCG-3′; Movement Protein (MP) (809 bp) [16] Forward primer, 5′-TAAGTTTGCTAGGTGTGATC-3′ and Reverse primer, 5′-ACATAGATGTCTCTAAGTAAG-3′; CGMMV RNA helicase subunit (1053 bp) [17] Forward primer, 5′-ATGGCAAACATTAATGAACAAAT-3′ and Reverse primer, 5′-AACCACACAGAAAACGTGGC-3′.

Visualisation of the conventional PCR products was performed using a 1% agarose gel, with amplified PCR products cleaned up with an Isolate II PCR and Gel Kit (Bioline Pty Ltd., Taunton, MA, USA). The purified PCR products were sequenced in both directions at the Australian Genome Research Facility Ltd. (Brisbane, QLD, Australia www.agrf.org.au (accessed on 2 October 2018)). Analysis was performed using Geneious^®^ (Biomatters Ltd, Auckland, New Zealand). (accessed on 5 October 2018)) and compared to submitted sequences on BLAST (https://blast.ncbi.nlm.nih.gov (accessed on 5 October 2018)). RT-qPCR was performed on a Rotor-Gene 6000 (Qiagen, Hilden, Germany) with SensiFAST^TM^ SYBR^®^ No-ROX One-Step Kit (Bioline Pty Ltd., Taunton, MA, USA) following the manufacturer’s conditions. The conditions used in this study can be found in a recent publication from the author [14]; the primers used were: Forward primer, 5′-GTGGTTTCTGGTGTATGGAACGTA-3′, Reverse primer, 5′-CGGGAGCTGAAAATTTGCATATAGT-3′, and probe (RZ_CGMMVmp-03), 5′-[FAM]CACCCCTACAGGATTC[NFQMGB]-3′ [18].

An internal plant control (NADH dehydrogenase ND2 subunit) [19] was undertaken to ensure quality assurance of sample extractions.

## 3. Results

### 3.1. Infectivity of Non-Host Plants Grown in Field and Screenhouse

Crops from the field trial, which included sorghum (*Sorghum bicolor*), sweetcorn (*Z. mays*), capsicum (*C. annum*), and peanut (*Arachis hypogaea*), were tested for their susceptibility to CGMMV. The individual crops were bulk sampled in batches of ten, and through RT-PCR and RT-qPCR, CGMMV was not detected in any of the sampled crops. For the screenhouse trial, crops which included those from the field trial, and the additional crops snake bean (*Vigna unguiculata* ssp. *sesquipedalis*) and okra (*A. esculentus*), were again tested for their susceptibility to CGMMV, in which the plants were mechanically inoculated, along with the addition of contaminated material to the soil in which they were planted. Each of the crops were bulked into eight samples of ten, in which all were tested through RT-PCR and RT-qPCR for the presence of CGMMV; no positive detections were observed in either RT-PCR or RT-qPCR testing (Table 1).

#### 3.1.1. Infectivity and Spread of CGMMV through Weeds

Testing of weed species inoculated with CGMMV revealed that a low number of plants were infected with the virus, and infection varied between species. Sabi grass, black nightshade, wild gooseberry, and fat hen were all difficult to determine the presence of viable virus, with only positive RT-qPCR results between them. The results of Amaranth revealed higher numbers of positive PCR results, returning positive results for both RNA Replicase Subunit and RT-qPCR (Table 2).

As pigweed bulk samples were negative for CGMMV using the RNA helicase Subunit RT-PCR but positive through RT-qPCR, further analysis was performed using the Coat protein and Movement protein (Table 3). Positive detections were observed in three of the eight bulk samples, with Sanger sequencing confirming the amplicon as CGMMV in one sample (Accession No. KM363234 and MH271419.1). The two remaining samples did not return readable sequences due to a high amount of mismatching.

#### 3.1.2. *Amaranthus viridis* as a Viable Host for CGMMV

Throughout the initial sampling period for CGMMV in 2015–2016, *A. viridis* was targeted routinely and was found to be positive for CGMMV at a higher rate than any other weed species. As a result, *A. viridis* was analysed further, with two leaves per plant collected from five pots, for a total of 10 leaves. The samples were then extracted and tested for the presence of CGMMV. In total, sixteen samples were tested for CGMMV, with 50% (8 of 16) testing positive in conventional RT-PCR tests and 75% (12 of 16) testing positive in RT-qPCR tests, with Ct values ranging from 19 to 33. Of the eight samples that tested positive to CGMMV using conventional RT-PCR, Sanger sequencing was able to confirm the amplicon as CGMMV in three samples (Accession No. KM363234 and MH271419.1) (Table 4).

Further analysis was performed, with individual pots of the three bulk samples where CGMMV was confirmed, and 15 pots sampled (pots 36–45 and 71–75) and tested individually for the presence of CGMMV. Pots 36, 39, and 74 tested positive to two or more RT-PCRs and the single RT-qPCR. However, the use of Sanger sequencing was unable to determine if CGMMV was present (Table 5).

To investigate the possibility of *A. viridis* transmitting CGMMV, bioassays were performed from selected bulk (samples 5, 6, 7, and 8) and individual (pots 36 and 39) samples. Material collected during the initial sampling and detection of CGMMV in the weeds was freeze dried for future use, with inoculum created from this material, and inoculated onto four watermelon plants per weed sample. At eight weeks post-inoculation, the watermelon samples were bulked together and tested for the presence of CGMMV. Of the four bulk samples, one bulk sample (bulk sample 7; pots 31–35) and the two individual *A. viridis* samples (pots 36 and 39) tested positive for CGMMV. Sanger sequencing confirmed the amplicon as CGMMV from samples 5 (pot 36) and 6 (pot 39) (Accession No. KM363234 and MH271419.1) (Table 6). Inoculated watermelon leaves were tagged to ensure these were not sampled.

## 4. Discussion

### 4.1. Susceptibility of Non-Host Plants

The selected non-host plants chosen for their susceptibility to CGMMV were found to be unsusceptible to the virus, regardless as to whether they were sown into infected soil (field) or had their leaves mechanically inoculated with CGMMV (screenhouse). Although the *Potyvirus* maize dwarf mosaic virus is known to infect sweetcorn [20], there are no known tobamoviruses that infect sweetcorn, which is evidenced by the inability of CGMMV to infect even when manually inoculated.

The *Tobamovirus* tobacco mosaic virus (TMV) is thought to be able to infect snake bean; however, there is little evidence from this study to suggest that CGMMV can infect this crop. This is likely due to TMV’s wide host range and CGMMV’s limited host range other than cucurbits [21].

In this study, okra was not able to be infected by CGMMV. This result follows previous research that was able to confirm that okra was not susceptible to the tobamoviruses TMV and pepper mild mottle virus (PMMoV) [22]. Few viruses are known to infect peanuts, and this study was able to confirm that CGMMV is not able to infect this host, with no detections in the field or screenhouse trials.

Although capsicum crops are susceptible to several tobamoviruses, including tomato mosaic virus (ToMV), TMV, and PMMoV, CGMMV was not able to cause infection in capsicum plants tested in the field and screenhouse tests. The inability of CGMMV to infect capsicum plants is likely due to the virus’s limited host range [20].

The inability of CGMMV to infect a range of non-host crops may allow for these crops to be grown on an infected property for a 12-month period in order to help disinfect the virus, with no host plants grown and weeds removed to prevent the replication of the virus. This may not only potentially allow for an alternative income, but also allow for cucurbit production to begin in uninfected/low titre CGMMV-infected soil.

### 4.2. Infectivity of CGMMV in Weeds

Although CGMMV was difficult to routinely detect in fat hen in this study, the weed is thought to be a host of the virus [3,11]; however, certain varieties of fat hen may be immune to the virus [23]. Initial testing of pigweed for CGMMV using the RNA helicase subunit resulted in negative results, with RT-qPCR indicating suspected positives in seven of the eight bulk samples. Further analysis with the Coat protein and Movement protein revealed that three of the eight samples were positive in both RT-PCRs, with Sanger sequencing confirming the virus as present in one of the samples. This result aligns with previous research indicating that pigweed is a host of CGMMV [11].

The ability of CGMMV to infect black nightshade was studied previously in 2004 [11], with indications that it may be a host of the virus; however, a more recent study in 2017 [3] revealed that there was little evidence of the virus being able to infect this weed species. The present study indicated that black nightshade may be a host of CGMMV; however, only RT-qPCR positives were observed, with Ct values of > 30.

Wild gooseberry has previously been shown to be susceptible to the *Tobamovirus* TMV [24]; there are, however, few indications that this weed is susceptible to CGMMV. The present study was able to demonstrate that wild gooseberry is not a host of CGMMV, with no RT-PCR and RT-qPCR positive results observed in 80 mechanically inoculated weeds. Although Sabi grass is susceptible to the *Potyvirus* JGMV [25], there are no known tobamoviruses that infect this species. Although there were positive RT-qPCR detections for CGMMV, there is little evidence to suggest that Sabi grass is a host of the virus, and the RT-qPCR results are likely to be false positives from host material or contamination from the inoculated leaves. The inability to routinely detect CGMMV within weeds may be a result of RNA interference (RNAi), in which sequence-specific sections of the viral genome are targeted and silenced, preventing systemic spread [26,27]. It is possible that within the weeds, there are higher numbers of Dicer-like proteins, Argonautes, and/or RNA-dependent RNA polymerases that can directly disrupt and block CGMMV reproduction and dissemination [21]. Further research into this area would be required.

### 4.3. A. viridis as a Weed Host of CGMMV

In a previous 2004 [11] study, two Amaranth species, *A. blitoides* and *A. retroflexus,* were found to be susceptible to CGMMV after manual inoculation, through ELISA and Immunocapture-RT-PCR. In a more recent study in 2017 [3], *A. viridis* weeds were collected from infected watermelon fields and CGMMV was not detected when tested via ELISA. In this study, *A. viridis* was found to be susceptible to CGMMV, as 8 of 16 bulk samples tested positive through at least two CGMMV-specific RT-PCRs, while 12 of 16 tested positive to a CGMMV-specific RT-qPCR. The inability of CGMMV to be detected by ELISA in the 2017 study [3] may be due to the distribution of CGMMV in the infected field, or a potential low titre of the virus that was undetectable by ELISA. It is also possible that CGMMV is not evenly distributed throughout *A. viridis,* potentially making detections of the virus difficult and sporadic.

To further analyse *A. viridis* as a host of CGMMV, four bulk samples and two single pots were used as an inoculum source on watermelon. One of the four bulk samples was deemed as a suspected positive for CGMMV, as the sampled watermelons produced positive RT-PCR and RT-qPCR (Ct 30) results. However, Sanger sequencing of the amplified amplicons was unable to confirm its presence. Watermelon plants inoculated with the two single samples (pot 36 and pot 39) tested positive for CGMMV through RT-PCR and RT-qPCR (Ct 24 and 25, respectively), and were confirmed positive via Sanger sequencing. In 2013 [28], weeds manually infected with tomato ringspot virus were able to serve as inoculum for tomato plants. More recently, a study observed the interactions between *P. angulate* and several cucurbit viruses, including watermelon mosaic virus and zucchini yellow mosaic virus. It was revealed that this weed was a host of several viruses, and served as a secondary source of infection for cucurbits [12].

The ability of weeds to be a source of inoculum for viruses, including CGMMV, has been highlighted in this study. The inability to consistently detect CGMMV in *P. oleracea*, *S. nigrum,* and *C. album* may indicate that these weeds are not an efficient source of inoculum, and may be a temporary reservoir. As watermelon plants were successfully infected using *A. viridis*-infected material, the removal and constant monitoring of weeds within cucurbit growing regions is crucial for the prevention and management of virus.

It is evident from this research, and through previous research, that further work must be undertaken to adequately review the interactions between CGMMV and potential weed hosts. Overcoming the difficulty of detecting the virus within the weeds may play a role in ultimately determining if it is systemically spread, and the risk it poses to cucurbit growing areas.

## Figures and Tables

**Table 1:** Non-host crops tested for their ability to be infected by cucumber green mottle mosaic virus.

Non-Host Crop	Season	Field Trial	Pot Trial
*Zea mays* (sweetcorn)	Dry	− ^1^	−
*Vigna unguiculata* ssp. *Sesquipedalis* (snake bean)	Dry	NA ^2^	−
*Capsicum annum* (capsicum)	Dry	−	−
*Abelmoschus esculentus* (okra)	Wet	NA	−
*Sorghum bicolor* (sorghum)	Wet	−	−
*Arachis hypogaea* (peanut)	Wet	−	−

^1^—Represents a negative or positive result. ^2^ NA = Not applicable, crops were not tested in this trial.

**Table 2:** RT-PCR (RNA Replicase Subunit) and RT-qPCR results of weeds manually infected with cucumber green mottle mosaic virus.

Non-Host Weeds	RT-PCR ^1^	RT-qPCR
*Amaranthus viridis* (Amaranth)	+ ^2^	+ (Ct 27) ^3^
*Portulaca oleracea* (pigweed) *	−	+ (Ct 26)
*Solanum nigrum* (black nightshade)	−	+ (Ct 31)
*Physalis angulata* (wild gooseberry)	−	−
*Chenopodium album* (fat hen)	−	+ (Ct 30)
*Urochloa mosambicensis* (Sabi grass)	−	+ (Ct 31)

^1^ RNA replicase subunit RT-PCR results. ^2^ −/+ Represents a negative or positive result. ^3^ Average Ct value of positive bulk samples. * Further testing of pigweed with other RT-PCRs resulted in positive detections of cucumber green mottle mosaic virus.

**Table 3:** Bulk *P. oleracea* pots tested for the presence of cucumber green mottle mosaic virus.

Sample (Pots)	RT-PCR ^1^	RT-qPCR
1 (Pots 1–10)	− ^2^	+ (Ct 29)
2 (Pots 11–20)	−	+ (Ct 26)
3 (Pots 21–30)	−	+ (Ct 26)
4 (Pots 31–40) *	+	+ (Ct 27)
5 (Pots 41–50)	+	+ (Ct 27)
6 (Pots 51–60)	+	+ (Ct 25)
7 (Pots 61–70)	−	−
8 (Pots 71–80)	−	+ (Ct 28)

^1^ RT-PCR represents results for both MP and CP, where both tests returned a positive result. ^2^ −/+ Represents a negative or positive result. * Sanger sequencing confirmed the presence of cucumber green mottle mosaic virus.

**Table 4:** Bulk *A. viridis* weeds tested for the presence of cucumber green mottle mosaic virus after mechanical inoculation.

Sample (Pots)	RT-PCR ^1^	RT-qPCR
1 (1–5)	− ^2^	−
2 (6–10)	−	+ (Ct 27)
3 (11–15)	+	+ (Ct 24)
4 (16–20)	−	−
5 (21–25)	+	+ (Ct 20)
6 (26–30) *	+	+ (Ct 21)
7 (31–35) *	+	+ (Ct 21)
8 (36–40) *	+	+ (Ct 19)
9 (41–45)	+	+ (Ct 21)
10 (46–50)	−	−
11 (51–55)	−	+ (Ct 29)
12 (56–60)	+	+ (Ct 22)
13 (61–65)	−	−
14 (66–70)	−	+ (Ct 33)
15 (71–75)	+	+ (Ct 30)
16 (76–80)	−	+ (Ct 24)

^1^ RT-PCR represents results for all three tests, where two or more tests returned a positive result. ^2^ −/+ Represents a negative or positive result. * Sanger sequencing confirmed the presence of cucumber green mottle mosaic virus.

**Table 5:** Individual *A. viridis* pots tested for the presence of cucumber green mottle mosaic virus.

Sample (Pots)	RT-PCR ^1^	RT-qPCR
1 (Pot 36) *	+ ^2^	+ (Ct 27)
2 (Pot 37)	−	−
3 (Pot 38)	−	+ (Ct 29)
4 (Pot 39) *	+	+ (Ct 31)
5 (Pot 40)	−	+ (Ct 31)
6 (Pot 41)	−	+ (Ct 33)
7 (Pot 42)	−	−
8 (Pot 43)	−	+ (Ct 33)
9 (Pot 44)	−	−
10 (Pot 45)	−	+ (Ct 28)
11 (Pot 71)	−	−
12 (Pot 72)	−	−
13 (Pot 73)	−	+ (Ct 28)
14 (Pot 74) *	+	+ (Ct 33)
15 (Pot 75)	−	−

^1^ RT-PCR represents results for all three tests, where two or more tests returned a positive result. ^2^ −/+ Represents a negative or positive result. * Sanger sequencing was unable to confirm the presence of cucumber green mottle mosaic virus.

**Table 6:** Watermelon plants inoculated with *A. viridis* positive material.

Sample (Pots)	RT-PCR ^1^	RT-qPCR
1 (Bulk Sample 5)	− ^2^	−
2 (Bulk Sample 6)	−	−
3 (Bulk Sample 7)	+	+ (Ct 30)
4 (Bulk Sample 8)	−	−
5 (Single Pot 36) *	+	+ (Ct 24)
6 (Single Pot 39) *	+	+ (Ct 25)

^1^ RT-PCR represents results for all three tests, where two or more tests returned a positive result. ^2^ −/+ Represents a negative or positive result. * Sanger sequencing confirmed the amplicon as cucumber green mottle mosaic virus.

## Data Availability

Not applicable.
